# Clinical Relevance of Specific Cognitive Complaints in Determining Mild Cognitive Impairment from Cognitively Normal States in a Study of Healthy Elderly Controls

**DOI:** 10.3389/fnagi.2016.00233

**Published:** 2016-10-04

**Authors:** Marina Ávila-Villanueva, Ana Rebollo-Vázquez, José M. Ruiz-Sánchez de León, Meritxell Valentí, Miguel Medina, Miguel A. Fernández-Blázquez

**Affiliations:** ^1^Alzheimer Disease Research Unit, CIEN Foundation, Carlos III Institute of Health, Queen Sofia Foundation Alzheimer CenterMadrid, Spain; ^2^Department of Basic Psychology II, Complutense University of Madrid (UCM)Madrid, Spain; ^3^CIBERNED (Network Center for Biomedical Research in Neurodegenerative Diseases)Madrid, Spain

**Keywords:** everyday memory questionnaire, factor analysis, item response theory, mild cognitive impairment, neuropsychological assessment, subjective cognitive complaints

## Abstract

**Introduction**: Subjective memory complaints (SMC) in the elderly have been suggested as an early sign of dementia. This study aims at investigating whether specific cognitive complaints are more useful than others to discriminate Mild Cognitive Impairment (MCI) by examining the dimensional structure of the Everyday Memory Questionnaire (EMQ).

**Materials and Methods**: A sample of community-dwelling elderly individuals was recruited (766 controls and 78 MCI). The EMQ was administered to measure self-perception of cognitive complaints. All participants also underwent a comprehensive clinical and neuropsychological battery. Combined exploratory factor analysis (EFA) and Item Response Theory (IRT) were performed to identify the underlying structure of the EMQ. Furthermore, logistic regression analyses were conducted to study whether single cognitive complaints were able to predict MCI.

**Results**: A suitable five-factor solution was found. Each factor focused on a different cognitive domain. Interestingly, just three of them, namely Forgetfulness of Immediate Information (FII), Executive Functions (EF) and Prospective Memory (PM) proved to be effective in distinguishing between cognitively healthy individuals and MCI. Based on these results we propose a shortened EMQ version comprising 10 items (EMQ-10).

**Discussion**: Not all cognitive complaints have the same clinical relevance. Only subjective complaints on specific cognitive domains are able to discriminate MCI. We encourage clinicians to use the EMQ-10 as a useful tool to quantify and monitor the progression of individuals who report cognitive complaints.

## Introduction

Subjective memory complaints (SMC) can be defined as a self-experienced persistent decline in memory or any other cognitive ability in comparison with a previously normal status. Regarding the elderly, the topic of SMC has been a focus of intense debate within the research literature during the past two decades. Perhaps, the reason for that is the clinical importance of SMC in predicting the onset of memory impairment and future dementia. A recent meta-analysis has shown that, independently of the objective memory performance, 6.6% and 2.3% of older people with SMC develop mild cognitive impairment (MCI) and dementia per year (Mitchell et al., 2014), respectively. Since there is increasing evidence that SMC may represent a very early manifestation of Alzheimer’s Disease (AD; Jessen et al., 2014), little is known about the clinical role of specific complaints on the transition between normal aging to cognitive impairment.

Although SMC increase with age, complaints tend to show only mild or non-significant correlations with objective memory performance. Instead, many cross-sectional studies have reported a close relationship between SMC and other subjective variables such as depression (Crane et al., 2007), anxiety (Comijs et al., 2002), perceived health (Montejo et al., 2014), personality (Pearman and Storandt, 2004) and quality of life (Montejo et al., 2011).

Structured questionnaires are considered the best approach of gaining insight into older adults’ SMC (Montejo et al., 2014). Basically, these questionnaires consist of a list of common memory failures that must be rated according to the frequency in which they are experienced by subjects. Although there are many questionnaires that have been proposed to evaluate SMC, the Everyday Memory Questionnaire (EMQ; Sunderland et al., 1984) is perhaps one of the most extended scales. The EMQ has been used to assess SMC in a variety of populations, including older adults (Garrett et al., 2010; Ossher et al., 2013). It consists of 28 items about memory failures that occur in everyday life. All items must be answered according to a Likert-type scale.

Despite the emphasis by Sunderland et al. (1984) about the unidimensionality of the EMQ, the analysis of its individual items evidences that, a high percentage of them do not exactly correspond to memory complaints. Rather, some items would involve various cognitive domains like, visual perception (“failed to recognize, by sight, close friends or relatives”), attentional processing (“been unable to follow the thread of a story”), language production (“found that a word is on the tip of your tongue”) or Executive Functions (EF; “forgotten a change in your daily routine”). This may be the reason for what several studies have reported the existence of various latent factors on the EMQ structure (Cornish, 2000; Royle and Lincoln, 2008; Calabria et al., 2011). In any event, investigations using the EMQ with older adults have exclusively focused on the overall score (Alegret et al., 2015), and have not addressed the role of the specific underlying factors upon differentiation between healthy controls and people with MCI.

This study aims at investigating whether specific cognitive complaints are more useful than others to discriminate MCI by investigating the underlying structure of EMQ’s items in a large community-dwelling older adult sample. We expect to find different cognitive complaints dimensions in the EMQ. Our secondary goals are, to propose a shortened version of EMQ based on discrimination and difficulty parameters of items within each factor, and to examine the ability of these specific dimensions to differentiate between MCI and healthy controls.

## Materials and Methods

### Participants

The participants of this study comprised 844 community-dwelling individuals aged 70 years and above. All of them were part of the Vallecas Project cohort, a community-based longitudinal investigation for early detection of AD. The Vallecas Project was launched by CIEN Foundation-Queen Sofia Foundation on October 2011 (Olazarán et al., 2015). The study was approved by the Research Ethics Committee of the Carlos III Institute of Health. Written informed consent was obtained by all the participants.

All participants underwent a detailed assessment protocol including past medical history, neurological and neuropsychological examination, as well as biochemical and genetic blood test. The complete visit was usually carried out within 4 h, with convenient breaks if necessary.

Every participant was independently diagnosed taking into account age, gender, cognitive reserve, functional information and neuropsychological scores. Cognitive diagnoses were agreed between neurologists and neuropsychologists at consensus meetings. In all cases, cognitively healthy subjects had to obtain a score of 0 in the global Clinical Dementia Rating (CDR; Hughes et al., 1982). Criteria from the National Institute on Aging-Alzheimer’s Association (NIA-AA) were used to diagnose MCI (Albert et al., 2011). A total of 766 individuals were classified as controls and 78 met the criteria for MCI.

### Subjective Complaints Assessment

We used the EMQ to measure cognitive complaints. This questionnaire was administered following the instructions provided in a previous Spanish validation study (Montejo Carrasco et al., 2012). Participants were asked to rate the 28 items according to the frequency with which they experienced each complaint. Items were scored on a 3-point scale, with 0 indicating “never, rarely”, 1 “occasionally, sometimes” and 2 “frequently, almost always”. Thus, the total score ranged from 0 to 56. In all cases, individuals completed the EMQ in the presence of a member of the research team.

### Neuropsychological Assessment

A comprehensive neuropsychological battery was applied by trained neuropsychologists in order to obtain information about visual perception, attention, memory, language, praxis and EF. A total of 10 cognitive tests were considered: Mini Mental State Examination (MMSE; Folstein et al., 1975); Clock Drawing Test; Free and Cued Selective Reminding Test (FCSRT; Buschke, 1984); Lexical and Semantic Verbal Fluency (Peña-Casanova et al., 2009); Forward and Backward Digit Span (Wechsler, 1997); Five Point Test (Lee et al., 1994); Rule Card Shifting Test (Wilson et al., 1996); Boston Naming Test (15-items version; Fernández-Blázquez et al., 2012); Imitation of Bilateral Postures and Symbolic Gesture (Peña-Casanova, 1990); and Digit Symbol Coding (Wechsler, 1997). In addition, the following three scales were also administered to collect further data with regard to functional performance and mood: Functional Activities Questionnaire (FAQ; Pfeffer et al., 1982), Geriatric Depression Scale (GDS; Yesavage et al., 1982) and State-Trait Anxiety Inventory (STAI; Spielberger et al., 1970).

### Data Analysis

Analyses were conducted using R version 2.15. (R Development Core Team, 2012). Differences between healthy controls and MCIs on baseline characteristics were evaluated with Mann-Whitney tests and Pearson’s *χ*^2^ as appropriate. To identify latent constructs in the structure of correlations among the 28 items of the EMQ, an exploratory factor analysis (EFA) was performed using exclusively control subjects. Since response categories were ordinal scores, a polychoric correlation matrix resulted in a preferable approach for EFA (Brown, 2006).

First, a descriptive analysis of items was developed in order to find out their individual distribution. Then, it was determined whether the assumptions of normality and sphericity were met. Since no prior theory exists regarding the structure of data, Weighted Least Squares (WLS) with oblique Promax rotation was selected as the factor extraction method. The procedure for determining the number of factors was Parallel Analysis. In addition to *χ*^2^, the most common indexes of goodness-of-fit, Root Mean Square Error of Approximation (RMSEA) and Standardized Root Mean Square Residual (SRMR) were used. Values no greater than 0.06 for RMSEA and lower than 0.08 for SRMR indicate acceptable fit (Hu and Bentler, 1999).

By means of an Item Response Theory (IRT) approach, we calibrated all retained items using single Graded Response Models (GRM), one for each factor. These kinds of models are the most appropriate to examine ordinal items, as well as they assume normality of the latent trait. GRM estimate a slope parameter and two location parameters for each 3-category item. After obtaining item’s parameters from the IRT calibration, we used this information to identify a shortened version of the EMQ that maintained adequate content coverage within each factor with maximum precision. To guide selection of items, we examined the item information functions of every single factor. Additionally, two quantitative criteria were established in order to produce the maximum amount of information (discrimination index >1) with optimal difficulty distribution (sum of location parameters ranged from 2 to 4). Therefore, those items that did not fulfill both criteria were excluded from their corresponding factor. Finally, internal consistency was estimated by means of Cronbach’s alpha coefficient for ordinal categories.

Since distribution of most variables and components of the EMQ did not fulfill all the assumptions for using parametric statistics, a Spearman correlation analysis was carried out between the resulting factors and demographic and cognitive variables. In addition, Mann-Whitney tests were to study differences between control and MCI groups. As proposed by Cohen (1988), non-parametric adjusted effect sizes were estimated through the approximation of the *z* distribution associated with the Mann-Whitney test. According to the value of *r*, a large effect is 0.5, a medium effect is 0.3 and a small effect is 0.1. Additionally, to facilitate the interpretation of results, measures of probability of superiority (PS) were also provided.

Finally, we also performed logistic regressions to examine whether age, education and gender along with the underlying EMQ’s factors were able to predict cognitive impairment. In order to measure the impact of the model upon data, a special consideration was given to tests of signification for the model estimators. Analysis of residuals and goodness-of-fit statistics were also performed to measure the degree of adjustment of the model to available data.

## Results

### Descriptive Analysis of the Sample

The sample consisted of 766 controls (90.8%) and 78 MCIs (9.2%). Demographic and cognitive data, as well as differences between both groups, are shown in Table 1. Significant differences were found for age and years of education in such a way that MCIs were older and less educated than controls. A larger percentage of males were also classified as MCI. Moreover, as expected, the majority of cognitive variables showed large differences in favor of controls (*p*-value < 0.001), except for trait anxiety, where no statistical differences were found.

**Table 1:** **Descriptive analysis and mean differences between control and MCI groups**.

	Control (*n* = 766)		MCI (*n* = 78)		*p*-value
	Mean	SD	Mean	SD
Age (years)	74.07	3.80	76.08	4.06	<0.001
Education (years)	11.15	6.69	8.04	6.00	<0.001
Sex	63% Female		50% Female		0.032
Cognitive performance
MMSE	28.75	1.46	26.09	2.28	<0.001
Clock drawing test	9.42	1.26	8.20	1.88	<0.001
FCSRT free immediate	24.90	5.73	13.89	5.26	<0.001
FCSRT total immediate	42.84	4.45	30.78	8.29	<0.001
FCSRT free delayed	9.90	2.49	4.50	2.83	<0.001
FCSRT total delayed	14.79	1.51	10.13	3.35	<0.001
Lexical verbal fluency	39.76	12.95	27.31	10.33	<0.001
Semantic verbal fluency	49.54	10.11	34.57	8.49	<0.001
Forward digits	7.40	1.87	6.37	1.55	<0.001
Backward digits	4.64	1.85	3.77	1.51	<0.001
Five point test	21.88	8.19	14.77	5.81	<0.001
Rule card shifting	3.02	3.10	7.11	3.10	<0.001
Boston naming test-15 items	12.83	1.85	9.69	3.07	<0.001
Posture imitation	7.27	1.20	6.31	1.26	<0.001
Symbolic gesture	9.70	1.00	9.47	0.96	0.001
Digit symbol coding	39.72	15.10	25.20	10.95	<0.001
FAQ	0.38	0.68	2.68	2.33	<0.001
GDS	1.47	2.17	2.73	2.78	<0.001
STAI state	14.51	8.80	17.76	10.97	0.037
STAI trait	16.77	9.68	17.00	9.57	0.887

*Note. MMSE, Mini-Mental State Examination; FCSRT, Free and Cued Selective Reminding Test; FAQ, Functional Activities Questionnaire; GDS, Geriatric Depression Scale; MCI, Mild Cognitive Impairment; SD, Standard Deviation; STAI, State-Trait Anxiety Inventory*.

### Exploratory Factor Analysis

First, descriptive statistics of individual items of EMQ were calculated (Table 2). Items 11, 19 and 27 were excluded from further analysis since their values of skewness and/or kurtosis were over |2.5|. Thus, a symmetrical distribution with the rest of 25 retained items was ensured to be applied to the EFA.

**Table 2:** **Descriptive statistics of individual items of everyday memory questionnaire (EMQ)**.

Items	Mean	SD	Median	Min	Max	Skew	Kurtosis
1	0.89	0.49	1	0	2	−0.23	0.79
2	0.39	0.54	0	0	2	0.97	−0.14
3	0.25	0.49	0	0	2	1.76	2.26
4	0.26	0.50	0	0	2	1.70	2.02
5	0.71	0.55	1	0	2	−0.01	−0.55
6	0.74	0.53	1	0	2	−0.16	−0.38
7	0.51	0.53	0	0	2	0.33	−1.16
8	0.61	0.56	1	0	2	0.23	−0.82
9	0.35	0.54	0	0	2	1.17	0.34
10	0.31	0.51	0	0	2	1.32	0.71
11	0.16	0.43	0	0	2	2.71	6.86
12	0.58	0.61	1	0	2	0.54	−0.63
13	0.89	0.50	1	0	2	−0.22	0.76
14	0.44	0.58	0	0	2	0.91	−0.17
15	0.48	0.55	0	0	2	0.59	−0.75
16	0.29	0.49	0	0	2	1.35	0.76
17	0.46	0.59	0	0	2	0.85	−0.27
18	0.21	0.42	0	0	2	1.66	1.41
19	0.09	0.33	0	0	2	4.02	16.63
20	0.37	0.53	0	0	2	0.99	−0.14
21	1.00	0.61	1	0	2	0	−0.32
22	0.34	0.53	0	0	2	1.22	0.47
23	0.52	0.62	0	0	2	0.79	−0.39
24	0.73	0.57	1	0	2	0.08	−0.51
25	0.26	0.50	0	0	2	1.71	2.06
26	0.54	0.61	0	0	2	0.66	−0.52
27	0.16	0.40	0	0	2	2.44	5.43
28	0.35	0.53	0	0	2	1.18	0.38

*Note. SD, Standard Deviation*.

The EMQ total score was not normally distributed (Shapiro-Wilk normality test, *W* = 0.93; *p* < 0.001). The mean of the EMQ total score was 13.18 ± 7.84 (range 0–47). We did not obtain significant association between EMQ and gender (*W* = 59,803; *p* = 0.55) nor age (ρ = 0.04; *p* = 0.28). Nevertheless, the correlation between EMQ total score and years of education was statistically significant (ρ = −0.16; *p* < 0.001), which meant that individuals with more years of education tended to report less cognitive complaints. The Cronbach’s alpha coefficient for the polychoric correlation matrix comprising the 25 items was 0.93.

Table 3 shows the factor loadings and the communalities and percentage of variance explained for the factors obtained. The measure of sample adequacy was appropriate for developing an EFA (KMO = 0.92; Barlett $\chi_{1035}^{2}$ = 4350.15; *p* < 0.001). Although the Parallel Analysis determined six dimensions as the optimal solution from a statistical point of view, we finally adopted an explanation with five components because it proved more reasonable in biological terms. The first factor corresponded to the items 2, 6, 8, 13, 15, 16, 17, 20, 21, 23 and 28 and explained 17% of the total variance. This component was called *Forgetfulness of Immediate Information (FII)*. The second component comprised the items 3, 4, 5, 9, 10 and 12 and explained 11% of the total variance; it was termed as *EF*. A third component, named as *Prospective Memory (PM)*, retained the items 7, 14, 18 and 22. Finally, the fourth and fifth factors comprised, respectively, items 1 and 24 and items 25 and 26. They were called *Forgetfulness of Common Objects (FCO)* and *Spatial Orientation (SO)*. The analysis of the polychoric correlation matrix by using Mardia’s tests revealed data to be reached a suitable multivariate normality (skew statistic = 7146.36 with *p* < 0.001; kurtosis statistic = 36.02 with *p* < 0.001). Likewise, reliability of all factors was considered appropriate.

**Table 3:** **Exploratory factor analysis (EFA) of the EMQ with component loadings of each item**.

	I	II	III	IV	V	Communalities
Item 21	**0.710**	−0.254				0.37
Item 8	**0.618**	0.193	0.211		−0.288	0.57
Item 20	**0.605**			−0.156		0.40
Item 13	**0.576**	−0.148	0.153			0.40
Item 15	**0.528**		0.347			0.54
Item 17	**0.502**	0.425	−0.236			0.56
Item 6	**0.502**			0.144		0.37
Item 16	**0.431**	0.267				0.52
Item 28	**0.339**		0.208	−0.107	0.109	0.31
Item 2	**0.328**	0.313	−0.108			0.40
Item 23	**0.312**		0.237	−0.176		0.26
Item 3	−0.106	**0.786**				0.53
Item 4	−0.249	**0.718**	0.221			0.51
Item 9	0.400	**0.437**	−0.155		−0.127	0.41
Item 10	0.125	**0.407**			0.179	0.37
Item 12	0.109	**0.383**			0.146	0.34
Item 5	0.211	**0.318**		0.282	−0.118	0.44
Item 18	0.180		**0.692**	−0.149		0.52
Item 22	0.252	0.182	**0.501**			0.61
Item 14	0.400		**0.430**		−0.127	0.62
Item 7	−0.123		**0.349**	0.328	0.188	0.42
Item 1			−0.128	**1.002**		0.85
Item 24	0.265	−0.220	0.120	**0.568**		0.60
Item 25	−0.150				**0.955**	0.91
Item 26	0.109		−0.105		**0.688**	0.51
Eigenvalue	4.230	2.760	2.000	1.670	1.650	
Proportion variance	0.170	0.110	0.080	0.070	0.070	
Cronbach coefficient	0.800	0.660	0.630	0.650	0.650	

*Bold values indicate retained items in each factor*.

### IRT Calibration

Preliminary non-parametric Mann-Whitney tests were carried out in order to ascertain whether the five components of the EMQ were able to distinguish between healthy controls and MCIs. Thereby, FII (*U* = 16,510; *p* < 0.001), EF (*U* = 17,176; *p* < 0.001) and PM (*U* = 19,932.5; *p* = 0.016) were found to differentiate between both groups. However, FCO (*U* = 24,588; *p* = 0.381) and SO (*U* = 23,920; *p* = 0.258) did not show relevant differences. Thus, according to the aims of this work, only FII, EF and PM were finally analyzed. FCO and SO were excluded for further analyses.

The parameter estimates from the three GRM calibrations are shown in Table 4. The slope values for all items ranged from 0.942 to 2.408, indicating a considerable variation in discrimination among them. However, items 14 and 22 showed the best discrimination for PM, while values for items of FII and EF were more homogeneous. On the other hand, despite the range of location parameters reflected a sizeable range of underlying cognitive complaints (−1.567 to 4.181), the majority of item response categories were selected by participants who had more complaints than average. These results pointed out that the items allowed to differentiate among individuals at the end of the complaints continuum.

**Table 4:** **Parameters estimated from the three graded response models (GRM)**.

	a	b1	b2
**FII**			
**Item 2**	1.320	0.547	3.361
Item 6	1.403	−0.820	2.765
**Item 8**	1.887	−0.245	2.470
Item 13	1.345	−1.451	2.373
**Item 15**	1.714	0.168	2.867
**Item 16**	1.816	0.787	3.107
**Item 17**	1.278	0.332	2.911
Item 20	1.353	0.601	3.444
Item 21	1.174	−1.567	1.542
Item 23	0.942	0.231	3.164
**Item 28**	1.115	0.302	3.681
**EF**			
**Item 3**	1.698	1.053	2.889
Item 4	1.473	1.076	3.100
Item 5	1.300	−0.805	2.792
Item 9	1.068	0.831	3.781
**Item 10**	1.238	0.315	3.612
**Item 12**	1.224	−0.075	2.703
**PM**			
Item 7	1.094	0.046	4.181
**Item 14**	2.273	0.327	2.203
Item 18	1.581	1.214	4.092
**Item 22**	2.408	0.594	2.456

*Note. a, discrimination parameter; b1, difficulty parameter response option 1; b2, difficulty parameter response option 2; AIC, Akaike Information Criterion; FII, Forgetfulness of Immediate Information; EF, Executive Functions; PM, Prospective Memory. AIC FFI: 11,292.92, AIC EF: 5938.43, AIC PM: 3742.51. Bold values are selected items for each factor*.

Then, we selected the best combination of items within each factor to maximize the amount of information with optimal difficulty distribution. We used both discrimination and difficulty parameters of items to carry out the selection. To that end, items should have discrimination indexes greater than one and location parameters ranged from 2 to 4. These criteria were adopted because of the objectives of this work (the easiest or the most difficult items were considered not advisable to discriminate between controls and MCI). Overall, 11 items were finally selected as follows: (i) FII: items 2, 8, 15, 16, 17 and 28; (ii) EF: items 3, 10 and 12; and (iii) PM: items 14 and 22. A final score of this shortened EMQ, called EQM-10, was also calculated by adding up these 11 items.

### Multivariate Study

**Figure 1:**
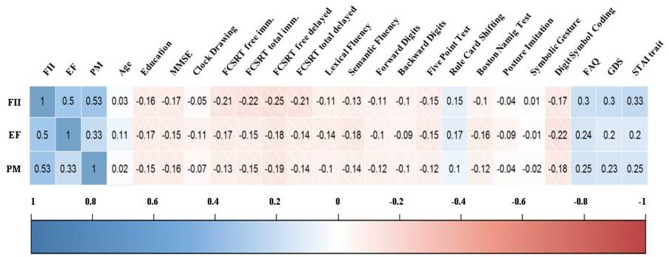
**Correlogram between the factors of the everyday memory questionnaire (EMQ) and demographic and cognitive variables**. Note. FII, Forgetfulness of Immediate Information; EF, Executive Functions; PM, Prospective Memory; MMSE, Mini-Mental State Examination; FCSRT, Free and Cued Selective Reminding Test; FAQ, Functional Activities Questionnaire; GDS, Geriatric Depression Scale; STAI, State-Trait Anxiety Inventory.

As shown in Figure 1, FII, EF and PM correlated among them in a range from 0.33 to 0.53. Regarding the neuropsychological tests, three factors showed low-moderate correlation coefficients with psychiatric symptoms, while the correlation with cognitive performance was mainly low. For depression and anxiety, the coefficients were positive, indicating that complaints increased as depression and anxiety scores were higher. On the contrary, the relationship with objective cognition showed negative coefficients, meaning larger complaints as cognitive performance decreased. Interestingly, FII was more associated with episodic memory (FCSRT), while EF was stronger related to language production (Fluency, BNT) and executive components (number of errors in RCS). Correlation coefficients between MMSE and every factor were very similar to those obtained in neuropsychological tests. Age and education showed low correlation coefficients with all factors.

**Figure 2:**
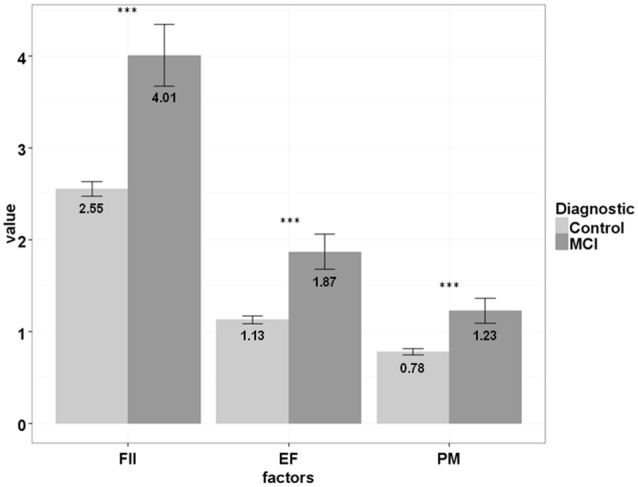
**EQM-10 factors scores differences between controls and MCIs**. Note. MCI, Mild Cognitive Impairment; FII, Forgetfulness of Immediate Information; EF, Executive Functions; PM, Prospective Memory. ^***^p-value < 0.001.

Non-parametric Mann-Whitney tests were performed to determine whether the three factors were able to distinguish between healthy controls and MCIs. Figure 2 shows the scores of both groups for each component. FII (*U* = 17,175; *p* < 0.001), EF (*U* = 17,651; *p* < 0.001) and PM (*U* = 19,015; *p* < 0.001) were found to differentiate between both groups. According to the non-parametric effect size, FII, EF and PM showed respectively the following sizes: 0.16, 0.14 and 0.12. Total score of the EMQ-10 was also significant (*U* = 14,098; *p* < 0.001) and showed a mild increase in the effect size (*r* = 0.18).

Finally, four logistic regression models were carried out to study the impact of cognitive complaints upon the diagnostic of MCI. All these five models were adjusted for age, education and gender as covariates (Table 5). The three cognitive factors proved to be significant in their respective models after controlling for demographic variables. In addition, estimates of all factors were positive, what indicated that expressing complaints was associated with MCI. FII showed the best deterministic coefficient (model 1; *R*^2^ = 0.14) followed by EF (model 2; *R*^2^ = 0.10) and PM (model 3; *R*^2^ = 0.10). Indeed, total score of EMQ-10 (model 4; *R*^2^ = 0.14) did not improve the association with diagnostic showed by FII. Hence, although the values of these determination coefficients were not too high, demographic variables and cognitive complaints were effective in distinguishing between cognitively healthy individuals and MCI.

**Table 5:** **Logistic regression models for diagnostic by EMQ-10’s factors and total score**.

Variables	B	SE	*z* value	Sig.
**Model 1: Age, Education, Sex and Forgetfulness of Immediate Information**
(Intercept)	−10.88	2.55	−4.28	<0.001
Age	0.12	0.03	3.71	<0.001
Education	−0.10	0.03	−3.68	<0.001
Female	−0.94	0.27	−3.50	<0.001
FII	0.24	0.05	4.78	<0.001
Null Deviance = 474.50 on 773 dg; Residual Deviance = 410.23 on 769 dg; AIC: 420.23
**Model 2: Age, Education, Sex and Executive Functions**
(Intercept)	−9.25	2.47	−3.75	<0.001
Age	0.10	0.03	3.13	0.002
Education	−0.09	0.03	−3.37	<0.001
Female	−0.70	0.27	−2.60	0.009
EF	0.34	0.10	3.49	<0.001
Null Deviance = 466.38 on 779 dg; Residual Deviance = 417.76 on 775 dg; AIC: 427.76
**Model 3: Age, Education, Sex and Prospective Memory**
(Intercept)	−10.88	2.49	−4.37	<0.001
Age	0.12	0.03	3.82	<0.001
Education	−0.07	0.02	−3.05	0.002
Female	−0.75	0.27	−2.83	0.005
PM	0.38	0.12	3.09	0.002
Null Deviance = 467.31 on 784 dg; Residual Deviance = 421.76 on 780 dg; AIC: 431.76
**Model 4: Age, Education, Sex and EQM-10**
(Intercept)	−11.43	2.62	−4.37	<0.001
Age	0.12	0.03	3.72	<0.001
Education	−0.08	0.03	−3.11	0.002
Female	−0.79	0.28	−2.82	0.005
EQM-10	0.15	0.03	4.78	<0.001
Null Deviance = 444.97 on 739 dg; Residual Deviance = 384.68.76 on 735 dg; AIC: 394.68

*All models adjusted for age, years of education and sex. Note. Dg, degrees of freedom; AIC, Akaike Informative Criterion; Objects; SE, Standard Error; FII, Forgetfulness of Immediate Information; EF, Executive Functions; PM, Prospective Memory*.

## Discussion

In the current study, we have examined the latent structure of the EMQ and the ability of specific cognitive complaints to differentiate between MCI and healthy controls. To that end, we analyzed a sample of 844 community-dwelling individuals over 70 years who voluntarily participated in a longitudinal investigation for early detection of AD. Of them, only the 766 control individuals were used to study the factor structure of the EMQ. To our knowledge, this is the first study that investigates the dimensional structure of the EMQ and compares how well specific cognitive complaints are able to discriminate MCI.

Our results highlight an adequate internal consistency of the EMQ, as well as a factorial structure. This outcome does not fit well with the Sunderland’s assumption on the unidimensionality of the questionnaire (Sunderland et al., 1984). Indeed, as already reported by other authors, the EMQ has proved to have an underlying structure composed of three (Calabria et al., 2011), four (Royle and Lincoln, 2008) or even five factors (Cornish, 2000). Rather than a specific questionnaire focused on memory complaints, the EMQ seems to be a more complex scale that is able to measure various domains of subjective cognitive impairment.

In our study, items 11 (failed to recognize, by sight, close friends or relatives), 19 (forgotten important details about yourself) and 27 (repeat to someone what you have just told them) were excluded from the EFA due to their skewed distribution. The reason for that exclusion may have to do with the fact that these three items seem to reflect severe symptoms which appear in mild dementia rather than in earlier stages (preclinical or prodromal phases). The final solution with the remaining 25 items comprised of a five-factor structure which explained up to 50% of EMQ’s total variance: (i) FII was associated with fails in immediate retrieval, as well as naming impairment; (ii) EF was related to distractibility, inhibition errors and monitoring; (iii) PM referred to things that someone has to recall in the next future; (iv) FCO had to do with forgetting personal details; and (v) SO was associated with difficulties for spatial orientating.

One crucial aim of the present study was to examine the association of SMC with neuropsychological performance and clinical diagnosis. EMQ’s factors exhibited higher correlation coefficients with psychiatric symptoms than with cognitive performance as other studies have already demonstrated (Balash et al., 2013). Global cognitive status assessed by means of MMSE was negatively correlated with all factors. In addition, as shown in Figure 1, FII and EF proved to be the factors that correlated higher with cognitive performance, especially episodic memory in the case of FII, and EF for EF. This outcome provides concurrent validity to the latent structure of the EMQ because the internal content of the factors is directly related to the cognitive domain supposedly assessed. Furthermore, the use of an IRT approach allowed us to find out the best 10-items that maximize the collection of information on cognitive complaints.

Regarding the clinical implications of this work, it has been suggested that cognitive complaints are able to distinguish between cognitively healthy elders and MCI (Buckley et al., 2013). In our study, three types of cognitive concerns are able to discriminate between controls and MCI. Higher scores in specific complaints on retrieval of immediate events, executive functioning and PM are related to prodromal stages of dementia. Indeed, our results indicate that their effect sizes give a PS of nearly 60%. That is, if two individuals, one control and one MCI, were selected at random, the score in any of these three factors would be higher for the MCI patient, 60% of the times. The fact that both forgetfulness of objects and SO do not show differences in control subjects and MCI could be explained because of the first of them refers to a high prevalent oversight in the elderly population (“Forgetting where you have put something”, “Forgetting where things are normally kept or looking for them in the wrong place”) and the other one is an idiosyncratic sign of mild dementia (“Getting lost or turning in the wrong direction on a journey, on a walk, or in a building where you have been before”). All these findings emphasize that not all cognitive complaints have the same clinical significance for prediction of cognitive impairment.

Concerning the limitations of the present study, the cross-sectional nature of our research is perhaps the most important one. Although our results suggest that specific cognitive complaints discriminate between controls and MCI, it remains unclear whether those specific complaints may be used to detect individuals at high risk of conversion to MCI in the future. Given that the Vallecas Project is still in progress, this is an important issue that shall be addressed in next visits. Another limitation is that family members of the participants were not available in all cases in order to confirm the severity of the cognitive complaints reported by subjects with MCI. This information could be very useful in future studies to minimize the effect of anosognosia, a common symptom in MCI that might bias the results to some extent. Finally, it should be desirable to study the link between cognitive complaints and other variables such as cognitive reserve that may influence on cognitive performance (Freret et al., 2015; Mondini et al., 2016). Since cognitive reserve has been positively related to both episodic and working memory (Lojo-Seoane et al., 2014), it could be hypothesized that self-perception of subjective deterioration could be increased in those individuals with low cognitive reserve.

In summary, not all cognitive complaints are effective in distinguishing healthy elderly individuals from those with MCI. Specific complaints related to episodic memory, EF and PM discriminate between controls and cognitively impaired subjects. Individuals who present these particular complaints and do not yet have a diagnosis of MCI may need special attention in terms of close clinical follow-up or an early cognitive intervention. The use of the EMQ-10 is highly recommended to quantify subjective decline and to monitor the longitudinal progression of individuals who report those cognitive complaints.

## Author Contributions

MÁ-V, AR-V, MV, MAF-B collected the data. All authors drafted the manuscript. MAF-B, JMR-SL, MÁ-V conducted the statistical analysis. All authors interpreted the data and critically edited the manuscript. All authors approve the submitted version of the manuscript and are accountable for the accuracy and integrity of the work.

## Funding

This work was supported by the CIEN Foundation and the Queen Sofía Foundation.

## Conflict of Interest Statement

The authors declare that the research was conducted in the absence of any commercial or financial relationships that could be construed as a potential conflict of interest.
